# Impact of the COVID-19 Pandemic on Cancer Death Locations in Japan: An Analysis of Excess Mortality Through February 2023

**DOI:** 10.2188/jea.JE20230235

**Published:** 2024-07-05

**Authors:** Shuhei Nomura, Marisa Nishio, Sarah Krull Abe, Akifumi Eguchi, Manami Inoue, Motoi Suzuki, Masahiro Hashizume

**Affiliations:** 1 Department of Health Policy and Management, School of Medicine, Keio University, Tokyo, Japan; 2 Division of Prevention, National Cancer Center Institute for Cancer Control, Tokyo, Japan; 3 Department of Global Health Policy, Graduate School of Medicine, The University of Tokyo, Tokyo, Japan; 4 Tokyo Foundation for Policy Research, Tokyo, Japan; 5 Department of Social Epidemiology, Graduate School of Medicine and School of Public Health, Kyoto University, Kyoto, Japan; 6 Center for Preventive Medical Sciences, Chiba University, Chiba, Japan; 7 Infectious Disease Surveillance Center at the National Institute of Infectious Diseases, Tokyo, Japan

**Keywords:** COVID-19, excess cancer mortality, cancer death place, end-of-life care

## Abstract

**Background:**

The novel coronavirus disease 2019 (COVID-19) pandemic has significantly impacted end-of-life decisions for cancer patients in Japan, with disparities existing between preferred and actual care settings. Our study investigates the potential shifts in cancer death locations during the pandemic and if there were excess cancer deaths.

**Methods:**

Utilizing national mortality data from the Ministry of Health, Labour and Welfare from January 2012 to February 2023, we identified cancer deaths using International Classification of Disease, 10^th^ revision codes. We assessed death locations, including medical institutions, nursing facilities, and homes. The Farrington algorithm was employed to estimate expected death counts, and the differences between observed and expected counts were denoted as excess deaths.

**Results:**

From January 2018 to February 2023, there was consistently increase in the weekly observed cancer deaths. The presence of a definitive excess during the pandemic period remains uncertain. The percentage of deaths in medical institutions declined from 83.3% to 70.1%, while home deaths increased from 12.1% to 22.9%. Between April 2020 and February 2023, deaths in medical institutions frequently fell below the 95% prediction lower limit. Home deaths consistently exceeded the 95% prediction upper limit, with significant excess deaths reported annually.

**Conclusion:**

Our study found a shift in cancer death locations from medical institutions to homes in Japan during the COVID-19 pandemic. Our study did not confirm an overall increase in cancer deaths during this period. As with global trends, the profound shift from hospitals to homes in Japan calls for a comprehensive exploration to grasp the pandemic’s multifaceted impact on end-of-life cancer care decisions.

## INTRODUCTION

The novel coronavirus disease 2019 (COVID-19) pandemic has profoundly impacted global healthcare infrastructures, introducing unparalleled barriers to medical services for patients, including those with cancer. In Japan, multiple sources indicate a significant reduction in cancer diagnoses and treatments during the pandemic’s apex.^1^^–^^3^ In response to the evolving situation and with the intent of ensuring public safety, the Japanese government initiated a policy in April 2020, recommending that medical institutions limit visitations, a measure that included terminally ill cancer patients.^4^

For cancer patients, the choice of their end-of-life care setting transcends logistical considerations; it profoundly affects their terminal experience.^5^ Research consistently demonstrates that a majority of these individuals harbor a preference for passing away at home.^6^ However, a marked disparity exists between this expressed desire and actual practice, with many unable to fulfill their wish to end their lives in their chosen environment.^5^ This incongruence is particularly salient in Japan. A 2017 national survey revealed that, given a hypothetical terminal cancer diagnosis, approximately 70% of participants would opt for their final moments at home.^7^ However, the majority of deaths predominantly transpire in medical institutions. Although there has been a decrease in hospital-related deaths post-2010 and an uptick in home deaths from 2019, by 2021, a substantial discrepancy persists: 65.9% of deaths occurred in hospitals, contrasted with a mere 17.2% at home.^8^

Amidst the turbulence of the COVID-19 pandemic, it is plausible that shifts in the locations of deaths, especially those related to cancer, may have occurred. Investigating these shifts and their implications is important. Such an inquiry holds profound significance for advancing global cancer treatment, more so in countries like Japan, characterized by a universal healthcare system and robust cancer screening and reporting mechanisms. With this background, our study embarks on an important objective: to assess if there were excess cancer deaths during the COVID-19 pandemic and to ascertain any consequential changes in the locations of these deaths.

## METHODS

### Study design and population

National data from January 2012 to February 2023 were obtained from the Ministry of Health, Labour and Welfare (MHLW) for a comprehensive time-series analysis. This study scrutinized variations in cancer-related mortality rates and their venues amid the COVID-19 pandemic.

### Data

Mortality records were extracted from the MHLW’s Vital Statistics system. All deceased individuals having a residence certificate in Japan, irrespective of nationality, were included. Exclusions were those with missing birth or residence data, those who died abroad, or transient residents without a residence card. The study focused on cancer-caused deaths (International Classification of Diseases, 10^th^ revision [ICD-10] codes C00–C97). The recorded death locations were grouped into seven categories: a) hospitals, b) clinics, c) recuperative hospitals or geriatric healthcare centers, d) midwifery hubs, e) nursing homes, f) homes, and g) other locations. In our analysis, based on previous studies,^9^ we reclassified these into: (A) all places, (B) medical institutions (a and b), (C) nursing facilities (c and e), and (D) homes (f).

### Statistical analysis

We utilized the quasi-Poisson regression model, commonly referred to as the Farrington algorithm.^10^^,^^11^ The expected fatalities for a specific week, t, were deduced from data spanning weeks t − w to t + w of years h − b to h + b. Here, w and b are specific parameters with h denoting the year corresponding to t, recognized as the reference duration.

Equation for quasi-Poisson regression in the Farrington model:

$$log(E(Y_{t}))=\alpha +\beta t+f^{T}(t)\gamma_{f(t)}$$

In this model, *Y_t_* signifies the death count for a particular week and is assumed to abide by a quasi-Poisson distribution having a specific dispersion value. The logarithm of the expected value of *Y_t_*, *log*(*E*(*Y_t_*)), is expressed as a linear combination of various factors. Specifically, *α* is the intercept, and *β* represents the linear time trend. The term $f^{T}(t)\gamma_{f(t)}$ embodies the seasonality effect. The superscript *T* on ***f***(*t*) indicates the transpose of the vector, ensuring that when multiplied with $\gamma_{f(t)}$ , the result is a scalar (inner product). Here, ***f***(*t*) is a vector containing values representing different time periods or seasons, and $\gamma_{f(t)}$ is a vector of regression coefficients corresponding to these periods. This structure allows the model to capture seasonal variations in the death count. To elucidate our approach to seasonality, we divided data from 1 year, which was not included in the reference period, into nine distinct periods, as highlighted in previous research methods.^10^^,^^11^

In this study, adhering to the methodology used by the United States Centers for Disease Control and Prevention,^12^ we estimated the expected number of deaths for a specific week by leveraging data from 3 weeks prior and 3 weeks subsequent to each week over the past 6 years (b = 6; w = 3). However, to ensure that data from the pandemic period does not influence the estimation of the expected number of deaths, this methodology intentionally omits data from a recent duration, equivalent to the number of weeks from 2020 up to the end of the study period (164 weeks in the case of our study). Note that this exclusion is consistent not just during the pandemic period, but throughout all research periods under consideration. A limitation of this methodology is its inability to capture the temporal trends within that period. For example, if there was an upward trend in deaths attributed to aging during this period, an easily observed excess might emerge, potentially pointing to the impacts of aging rather than the pandemic.

Considering this limitation, we conducted a sensitivity analysis incorporating data from the pandemic period into the estimation of expected deaths. We used data from 3 weeks before and after each week (w = 3) over the past 5 years (b = 5), meaning for the 2022 estimation, we employed data from 2017 to 2021. Through the application of Anscombe residuals, the Farrington algorithm was adjusted to account for outliers during the pandemic (eg, excesses) by providing a weighting system to dampen their impact.^11^ This methodology is also commonly employed in excess death analyses, with the settings for b and w being consistent with prior studies.^9^^,^^13^^,^^14^

The estimated expected number of deaths was calculated using 95% two-sided prediction intervals for both the upper and lower bounds. The difference between the observed and expected death count was utilized to compute the number of excess deaths. We defined the percent excess as a relative measure of the magnitude of the excess, formulated as

$$\text{Percent excess mortality}=\left(\frac{\text{observed}-\text{expected}}{\text{expected}}\right)\times 100$$

Based on the definition of a week as specified in the Infectious Diseases Weekly Report by the National Institute of Infectious Diseases, daily data were transformed into weekly data. All analyses and graphical outputs were executed using R version 4.1.0 (R Foundation for Statistical Computing, Vienna, Austria). The Farrington algorithm was analyzed using the R package “surveillance”.^15^

## RESULTS

From January 2018 to February 2023, the observed number of deaths due to cancer was as follows: 374,640 in 2018 (over 52 weeks, averaging 7,204.6 per week), 377,366 in 2019 (over 52 weeks, averaging 7,257.0 per week), 386,014 in 2020 (over 53 weeks, averaging 7,283.3 per week), 382,647 in 2021 (over 52 weeks, averaging 7,358.6 per week), 387,172 in 2022 (over 52 weeks, averaging 7,445.6 per week), and 59,559 in 2023 (over 8 weeks, averaging 7,444.9 per week) (Table 1). The proportions of deaths in medical institutions, nursing facilities, and homes in 2018 were 83.3%, 3.9%, and 12.1% respectively, but by 2023, these proportions shifted to 70.1%, 7.1%, and 22.9%. Weekly trends of observed deaths since 2012 can be seen in eFigure 1.

**Table 1.** Observed and excess deaths by location from January 2018 through February 2023

Year	2018	2019	2020	2021	2022	2023
All places						
Observed	374,640	377,366	386,014	382,647	387,172	59,559
Excess	−9,618	−1,115	−1,345	3,437	7,955	−242
Percent excess, median (IQR)	−2.5 (2.3)	−0.1 (1.8)	−0.2 (2.0)	1.0 (3.2)	2.0 (2.1)	−0.1 (2.4)
Medical institutions						
Observed	311,904	312,069	299,317	275,829	272,865	41,723
Excess	−8,307	−1,295	−15,837	−28,750	−30,700	−6,572
Percent excess, median (IQR)	−2.6 (2.3)	−0.4 (2.2)	−5.3 (4.0)	−9.7 (2.1)	−10.4 (2.9)	−13.4 (2.7)
Nursing facilities						
Observed	14,688	16,215	18,501	21,417	25,146	4,235
Excess	−2,226	−2,272	−994	2,219	3,557	636
Percent excess, median (IQR)	−13.7 (6.9)	−12.8 (9.8)	−3.5 (12.0)	11.5 (10.8)	16.1 (8.8)	19.6 (9.1)
Homes						
Observed	45,249	46,487	65,184	82,040	85,580	13,048
Excess	−1,044	−913	12,339	27,323	31,223	5,130
Percent excess, median (IQR)	−1.3 (6.4)	−2.5 (7.6)	30.2 (26.5)	48.4 (10.9)	57.3 (8.6)	64.4 (8.3)

IQR, interquartile range.

2018 = January 1, 2018 through December 30, 2018 (52 weeks); 2019 = December 31, 2018 through December 29, 2019 (52 weeks); 2020 = December 30, 2019 through January 3, 2021 (53 weeks); 2021 = January 4, 2021 through January 2 (52 weeks), 2022; January 3, 2022 through January 1, 2023 (52 weeks); 2023 = January 2, 2023 through February 26, 2023 (8 weeks).

**Figure 1.**
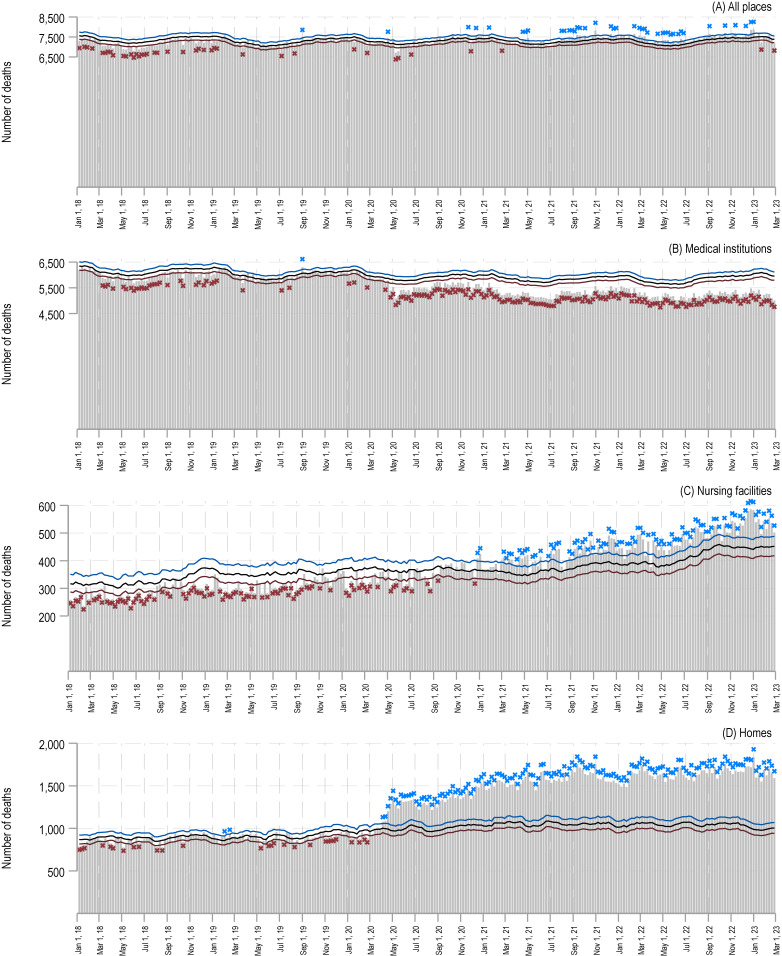
Excess cancer death trends by location from 2018–2023. This figure displays the weekly excess death count from January 2018 to February 2023, categorized by the death location: (**A**) all places, (**B**) medical institutions, (**C**) nursing facilities, and (**D**) homes. The blue and red lines depict the 95% upper and lower expected death count limits. Weeks where deaths surpass the 95% upper limit are marked with a blue cross, while those falling below the 95% lower limit are highlighted in red. Please note that the y-axis range varies across panels.

Figure 1 presents the observed weekly deaths, along with the projected numbers and their respective 95% upper and lower prediction intervals. Starting in May 2021, for ‘all places’, there are sporadic weeks where observed deaths exceed the 95% prediction upper limit. However, upon examining the results of the sensitivity analysis (eFigure 2), such weeks are largely absent. A similar trend is observed for nursing facilities. Before the pandemic, observed weekly deaths for both all places and nursing facilities often fell below the 95% prediction lower limit, but this is not evident in the sensitivity analysis. This can be attributed to the methodology used. As described in the methods section, the Farrington’s method excludes the most recent 164 weeks of data when estimating the baseline. Thus, any trend changes within this period would not be considered in the projection, possibly resulting in discrepancies between the predicted and observed values.

**Figure 2.**
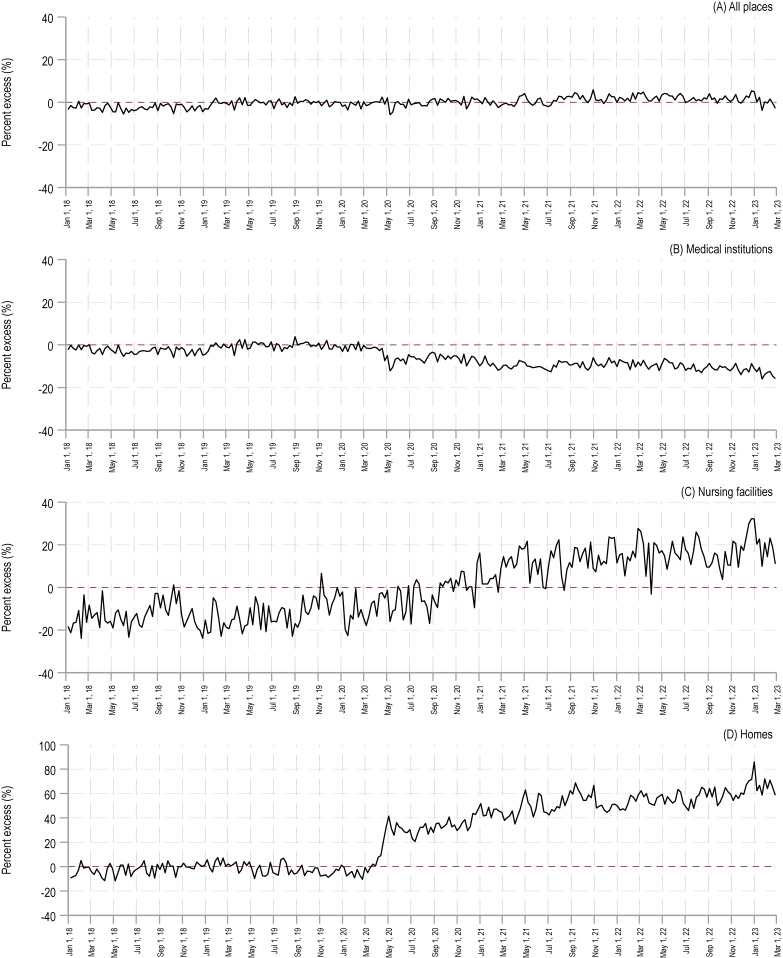
Percentage excess of weekly cancer deaths by location from 2018–2023. This figure presents the weekly percentage excess in cancer deaths from January 2018 to February 2023, categorized by the death location: (**A**) all places, (**B**) medical institutions, (**C**) nursing facilities, and (**D**) homes.

For medical institutions, from April 2020 to February 2023, observed deaths consistently fell below the 95% prediction lower limit. Notably, April 2020 is when Japan first declared a nationwide state of emergency. The median weekly percent excess was −5.3% in 2020 (interquartile range [IQR], 4.0%), −9.7% in 2021 (IQR, 2.1%), −10.4% in 2022 (IQR, 2.9%), and −13.4% in 2023 (IQR, 2.7%) (Figure 2).

Conversely, for homes, the trend is opposite that of medical institutions. From April 2020 to February 2023, observed deaths consistently exceeded the 95% prediction upper limit. The median weekly percent excess was 30.2% in 2020 (IQR, 26.5%), 48.4% in 2021 (IQR, 10.9%), 57.3% in 2022 (IQR, 8.6%), and 64.4% in 2023 (IQR, 8.3%) (Figure 2).

## DISCUSSION

In this study, a significant shift was observed in the location of cancer deaths from medical institutions to homes in Japan, yet there was no evidence of an increase in cancer deaths associated with the pandemic. This shift parallels findings in England, where Pring et al found a similar trend with a particularly prominent increase in home deaths among the Asian population.^16^

Various factors might explain this shift in death locales. First, the pandemic-induced constraints on healthcare visitations could have spurred an increase in home discharges. Japan’s novel coronavirus policy, launched in April 2020, advised curtailing non-urgent facility visits to hinder infection spread.^4^ Such a policy might have driven patients and their kin to lean towards home care. This directive could have incentivized patients and their families to favor home-based care. There exists an urgent requirement to probe this trend further to ascertain if these patients indeed procured their desired quality of care, particularly for those desiring to pass in familial surroundings within hospital environments.

Another consideration is the diffusion of Advance Care Planning (ACP). ACP focuses on preparing people and their families for medical decision-making, especially at the end-of-life.^17^ With the pandemic casting a shadow on mortality perceptions and due to mobility restrictions leading to prolonged family time, there has possibly been an uptick in ACP discussions. Japan’s own “Jinsei-kaigi (Life Conferences)” initiative, which fosters dialogues about terminal care decisions within families, might have inadvertently bolstered the trend towards home deaths.^18^

Financial implications also warrant attention. The pandemic has compounded the financial strain cancer patients and their families face. Economic challenges emerging from temporary work stoppages during treatment, and even permanent exit from the workforce, become more pronounced in the face of the pandemic.^19^^–^^21^ The disparity between the costs of home care and that of institutional medical care might have led those with economic vulnerabilities to opt for the former.^22^ A resultant concern is a potential decline in end-of-life care quality. It is vital that subsequent research endeavors scrutinize this economic disparity’s evolution and its specific manifestations.

As for the lack of clear indications of excess cancer deaths, the relatively brief observational period of merely 3 years post-pandemic might be a contributory factor. The observational window may not have adequately captured the comprehensive implications. Another hypothesis suggests that the reduction in cancer diagnoses was merely transient^2^ and may not have been significant enough to induce excess cancer deaths. Our findings align with those reported by Tanaka et al (2023).^23^ They analyzed the mortality rates of malignant neoplasms (ICD-10 codes C00–C96) up to 2021, standardized based on the 2015 population. Despite cancer patients having to postpone non-urgent surgeries, suspend outpatient treatments, and modify treatment methods, they reported that the mortality rate in 2021 was comparable to the pre-COVID era, and in 2020, it was notably lower than previous years.

While our findings provide valuable insights, they are some limitations. First, we only evaluate changes in the number of cancer deaths. We do not assess changes in the incidence of cancer, and thus cannot determine any change in the case fatality rate of such patients. The COVID-19 pandemic has brought about extensive changes in health-seeking behaviors, so any excess or deficit in cancer deaths may not accurately reflect changes in disease incidence.

Second, as described in the methods, the Farrington algorithm, depending on whether it incorporates data from the pandemic period for predicting expected deaths, can be significantly influenced by the presence of any excess or deficit in the period, even if this is adjusted for using Anscombe residuals. The former can capture temporal trends during the period but may not ignore the effects of outliers (excess or deficit) from the pandemic. The latter need not be concerned with these outliers but cannot account for recent data and temporal trends in the interim. For example, as shown in eFigure 1, there seems to be a slight shift from an increasing trend to a decreasing trend in the observed death counts in nursing facilities around 2017. Using the latter approach, this decrease was not taken into account in our baseline estimation for a period after 2017, specifically for 2018 and 2019, due to the Farrington algorithm excluding the most recent 164 weeks. As a result, this led to a higher estimated value, resulting in the detection of negative excess deaths in 2018 and 2019 in Figure 1. In contrast, employing the former approach was able to capture the trend shift around 2017, resulting in no noticeable underestimation of deaths between 2018 and 2019, as demonstrated in eFigure 2. Furthermore, to cite another instance, as can be observed in the ‘homes’ panel of eFigure 2, using the former approach led to a problem where, influenced by the continued excess post-2020, point estimates commenced an evident increasing trend around 2021. However, when employing the latter approach that prevents inclusion of data during the pandemic in the baseline, no such trend in point estimates was observed in the ‘homes’ panel of Figure 1, intuitively providing a more accurate assessment of the impacts associated with the pandemic. Arguing which is correct is not appropriate; it is a matter of choice, and it is essential to consider the nature of each and to take a holistic view of the results from both approaches.

In conclusion, the COVID-19 pandemic’s impact goes beyond immediate health threats, influencing cancer death trends. The evident shift from hospital settings to homes in Japan, mirroring global patterns, demands further thorough exploration to grasp its extensive implications.

## SUPPLEMENTARY MATERIAL

eFigure 1. Weekly trends in the number of cancer deaths by location from 2012–2023. This figure illustrates the weekly trends in the number of deaths from January 2012 to February 2022, categorized by the place of death: (**A**) all places, (**B**) medical institutions, (**C**) nursing facilities, and (**D**) homes.

eFigure 2. Excess cancer death trends by location from 2018-based on the sensitivity analysis. This figure displays the weekly excess death count from January 2018 to February 2023, sorted by the death location: (**A**) all places, (**B**) medical institutions, (**C**) nursing facilities, and (**D**) homes. The blue and red lines depict the 95% upper and lower expected death count limits. Weeks where deaths surpass the 95% upper limit are marked with a blue cross, while those falling below the 95% lower limit are highlighted in red.
