# Supplementary material for: Impact of the COVID-19 Pandemic on Cancer Death Locations in Japan: An Analysis of Excess Mortality Through February 2023
Source: J Epidemiol. 2024 Jul 5;34(7):349–55. doi: 10.2188/jea.JE20230235 (PMC11167266; doi:10.2188/jea.JE20230235)

eFigure 1. Weekly trends in the number of cancer deaths by location from 2012–2023. This figure illustrates the weekly trends in the number of deaths from January 2012 to February 2022, categorized by the place of death: (**A**) all places, (**B**) medical institutions, (**C**) nursing facilities, and (**D**) homes.

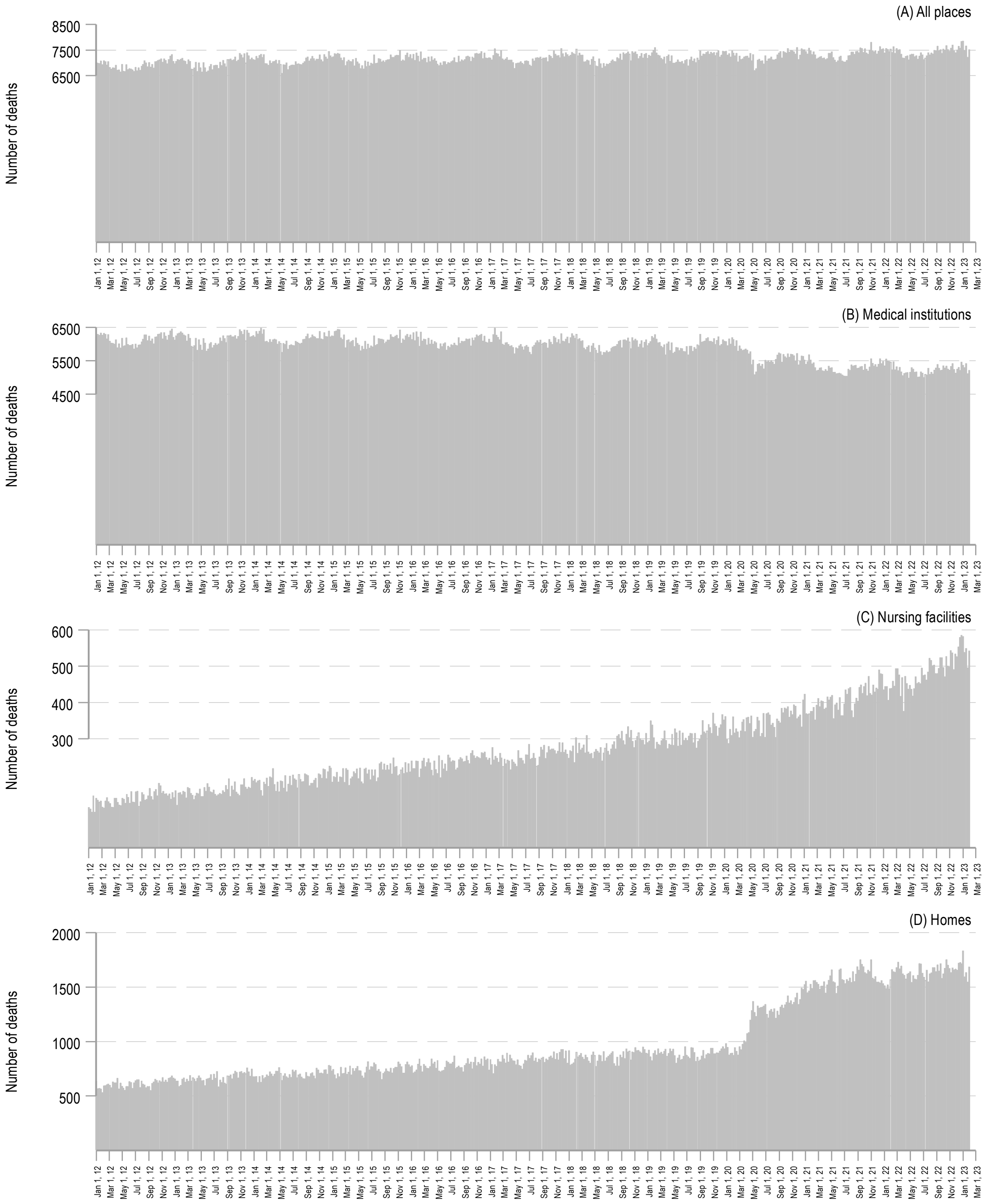

eFigure 2. Excess cancer death trends by location from 2018- based on the sensitivity analysis. This figure displays the weekly excess death count from January 2018 to February 2023, sorted by the death location: (**A**) all places, (**B**) medical institutions, (**C**) nursing facilities, and (**D**) homes. The blue and red lines depict the 95% upper and lower expected death count limits. Weeks where deaths surpass the 95% upper limit are marked with a blue cross, while those falling below the 95% lower limit are highlighted in red.

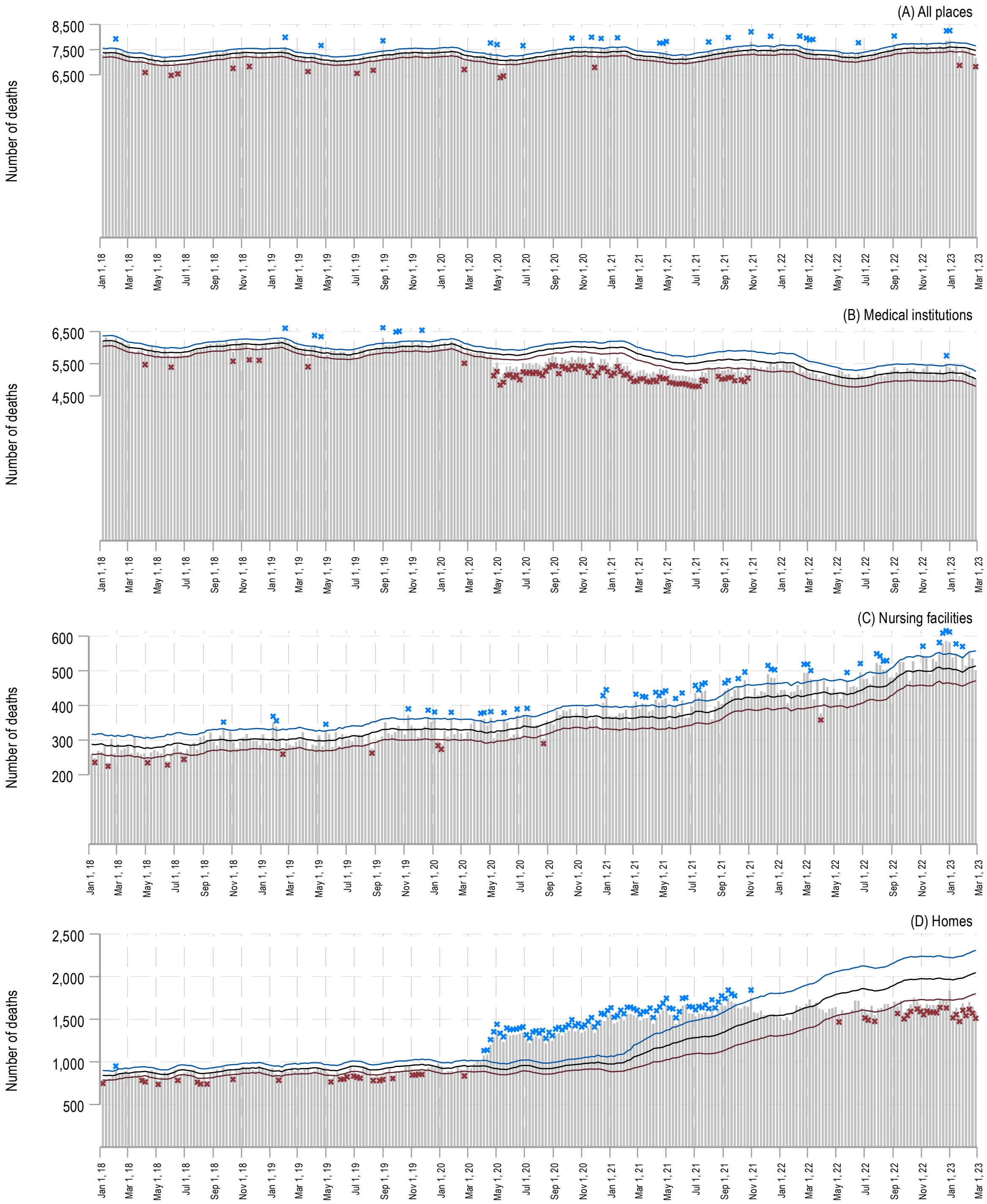

Supplement: Supplementary file 1 [file je-34-349-s001.pdf]
